# Oral hygiene and caries experience in children with down syndrome and autism spectrum disorder: a systematic review and meta-analysis

**DOI:** 10.3389/fdmed.2025.1726952

**Published:** 2026-01-23

**Authors:** Nurzhamal Bainazarova, Kuralay Zhumabayeva, Gulzhan Yermukhanova, Yagut H. Hajiyeva, Kulmira Abdykerimova, Lyazat Orakbay, Farida Zhumageldiyeva, Indira Karibayeva, Nurlan Jainakbayev

**Affiliations:** 1 Kazakhstan-Russian Medical University, Almaty, Kazakhstan; 2 Asfendiyarov Kazakh National Medical University, Almaty, Kazakhstan; 3 Azerbaijan Medical University, Baku, Azerbaijan; 4 University of Michigan Michigan Medicine, Ann Arbor, MI, United States; 5 Georgia Southern University, Statesboro, GA, United States

**Keywords:** autism spectrum disorder, DMFT, down syndrome, gingival index, oral health, oral hygiene index, plaque index

## Abstract

**Introduction:**

Children with Down syndrome (DS) and autism spectrum disorder (ASD) are at increased risk for oral health problems due to anatomical, behavioral, and socioeconomic factors. However, evidence on their caries experience and oral hygiene remains inconsistent. This study systematically reviewed and meta-analyzed case–control and cross-sectional studies comparing oral health indices in children with DS or ASD to neurotypical peers.

**Methods:**

A systematic search was conducted in PubMed, Web of Science, Science Direct, and Google Scholar using a standardized strategy. Eligible studies included children aged 0–18 years. Pooled mean differences (MD) in Plaque Index (PI), Gingival Index (GI), DMFT (decayed, missing, and filled permanent teeth), dmft (primary teeth), and Simplified Oral Hygiene Index (OHI-S) with 95% confidence intervals (CI) were calculated in R using meta and metafor packages.

**Results:**

Twenty-four studies were included (527 children with DS, 1,221 with ASD, 1,875 controls). For PI, children with DS had MD = 0.53 (95% CI: −0.13–1.18; *I*^2^ = 90%) and children with ASD 0.28 (95% CI: −0.05–0.61; *I*^2^ = 93.3%) compared to controls. GI was MD = 12.10 (95% CI: −0.14–162.92; *I*^2^ = 99.7%) for DS and 0.33 (95% CI: −0.13–0.78; *I*^2^ = 93.1%) for ASD. DMFT showed MD = –0.29 (95% CI: −0.97–0.39; *I*^2^ = 54.7%) for DS and 0.29 (95% CI: −0.53–1.11; *I*^2^ = 97.6%) for ASD. dmft was MD = –0.14 (95% CI: −0.61–0.33; *I*^2^ = 0%) for DS and −0.33 (95% CI: −1.49–0.82; *I*^2^ = 94.6%) for ASD. OHI-S was MD = 0.28 (95% CI: −0.92–1.47; *I*^2^ = 92.2%) for DS and 0.31 (95% CI: −1.37–1.98; *I*^2^ = 65.7%) for ASD. Most differences were not significant due to high heterogeneity. Sensitivity analysis identified one influential study affecting PI; excluding it strengthened the effect (MD = 0.43; 95% CI: 0.17–0.70; *p* = 0.0047). No publication bias was detected for DMFT and dmft indices. Overall certainty of evidence was low.

**Conclusions:**

Children with DS and ASD showed no consistent differences in PI, GI, DMFT, dmft, or OHI-S scores compared to neurotypical peers. Public health strategies should focus on inclusive oral health education, provider training, and equitable access to dental services to improve outcomes for children with neurodevelopmental disorders.

**Systematic Review Registration:**

https://www.crd.york.ac.uk/prospero/display_record.php?ID=CRD420251155866, identifier: CRD420251155866.

## 1 Introduction

Down syndrome (DS) and Autism Spectrum Disorder (ASD) represent two of the most prevalent neurodevelopmental conditions in children. According to the World Health Organization, DS occurs in approximately 1 out of every 1,000–1,100 live births (1), whereas ASD is currently identified in about 1 in 100 children (2). Both are associated with lifelong cognitive, behavioral, and health challenges that require substantial healthcare resources (3–5). Overall, children with disabilities have poorer health than their non-disabled peers, and studies consistently show health disparities between these groups (6). In this context, children with DS and ASD have higher rates of oral health problems than typically developing peers (7, 8). A meta-analysis reported a high burden of oral diseases among children with ASD, with pooled prevalence estimates of 60.6% for dental caries and 69.4% for periodontal disease (9).

Children with DS often have a smaller midface and breathe through the mouth (10). Typical oral findings are open bite, a large-appearing tongue, cracks on the tongue and lips, late tooth eruption, small or misshapen teeth, missing teeth, a poor bite, and crowded teeth (11–14). These factors contribute to an increased risk of dental caries, periodontal disease, inflammation, and plaque accumulation (15, 16). Although ASD does not directly cause oral disease, affected individuals have elevated oral-health risk due to communication barriers, atypical sensory processing, and behavioral challenges that limit cooperation with hygiene and treatment (17, 18).

Moreover, children with neurodevelopmental disorders incur higher healthcare costs than their peers (19); dental care is often costlier due to frequent use of behavior management, sedation, or general anesthesia (20). A survey that included 40,840 children with special health care needs (SHCN) and 6,113 children without SHCN shows that they represent about 8.8 million and 61 million children nationally. Among children with SHCN, 81% needed preventive dental care and 24% needed other dental care in the past 12 months (21).

However, the literature regarding the oral health status of children with DS and ASD is conflicting and inconclusive. While some studies have reported higher rates of caries and plaque accumulation (22, 23), some investigations have paradoxically indicated that children with DS and ASD may exhibit fewer carious lesions and reduced dental plaque (24–26), and healthier gingival conditions (27) compared to their healthy counterparts.

In the existing literature, several systematic reviews and meta-analyses have assessed the oral health status of individuals with DS and ASD, including pediatric populations. With respect to DS, Martins et al. (25) conducted a systematic review and meta-analysis entitled “*The Incidence of Dental Caries in Children with Down Syndrome*,” which evaluated dental caries using the DMFT/dfmt indices and included studies published up to December 2020**.** In contrast, Scalioni et al. (28) performed a systematic review focusing on periodontal disease in patients with DS, with database searches conducted through March 2017**; this** review predominantly included case–control studies and did not undertake a quantitative meta-analysis. Furthermore, Deps et al. (29), in their systematic review and meta-analysis “*Association between Dental Caries and Down Syndrome*,” applied broader inclusion criteria that encompassed mixed-age populations, including individuals older than 18 years, thereby limiting the specificity of conclusions applicable to children.

Regarding ASD, Lam et al. (30) conducted a systematic review and meta-analysis examining the oral health status of children and adolescents with ASD, restricting inclusion to case–control studies published up to March 2018**.** Similarly, Silvana Nunes da Silva et al. (9), performed a systematic review and meta-analysis titled “*Oral health status of children and young adults with autism spectrum disorders*,” which evaluated oral health outcomes in individuals with ASD; however, the inclusion of young adults aged 18 years and older alongside pediatric populations constrains the applicability of findings specifically to children.

This inconsistency likely stems from non-standard outcome definitions**,** mixing of age groups**.** As a result, we still lack clear, comparable, age-stratified, and severity-focused estimates of oral health in these populations. Therefore, there is a need for an updated systematic review and meta-analysis that focuses exclusively on pediatric populations, applies standardized oral health outcome measures, and accounts for methodological heterogeneity across studies.

Understanding oral health status among children DS and ASD is essential for effectively promoting their oral health. The hypothesis guiding this review is that children with DS and ASD experience significantly poorer oral health outcomes than their healthy counterparts. This study aims to systematically review the literature and conduct meta-analysis to address the evidence gap, involving children aged 0–18 years with DS or ASD that report standardized outcomes.

## 2 Materials and methods

### 2.1 Study registration

The review protocol was submitted to PROSPERO (ID: CRD420251155866) on 26 September 2025, after confirming that no similar reviews existed.

### 2.2 Search strategy

Before conducting the comprehensive search the PROSPERO data base was searched, and no similar reviews were found.

The following databases were searched: PubMed, Web of Science, Science Direct, Google Scholar, Other sources, including manual searching of references (Table 1). Filters applied included publication in English and Russian, publication in scholarly journals, and document types limited to articles, research articles, and early access articles. No restrictions were placed on the year of publication.

**Table 1:** Search strategy used for the systematic review.

Database	Search Fields	Filters
PubMed	Title, Abstract, Keywords	Language: English, Russian
		Human
Web of Science	Title, Abstract, Keywords	Language: English, Russian
		Source Type: Articles
ScienceDirect	Title, Abstract, Keywords	Language: English
		Document Type: Articles
Google Scholar	Title, Abstract, Keywords	Language: English
		Document Type: Articles

To define the search terms, a preliminary search was conducted in PubMed to identify relevant keywords from the titles and abstracts of studies focusing on the prevalence of oral health conditions specifically oral hygiene, dental caries, and related indices among pediatric populations with DS and ASD.

Based on this initial exploration, the following keywords were used in the final search strategy: “oral health” OR “oral hygiene” OR “dental caries” OR “tooth decay” OR “plaque index” OR “gingival index” OR “OHI-S” OR “DMFT” OR “dmft” OR “Down Syndrome” OR “Trisomy 21” OR “Autism Spectrum Disorder” OR “autistic children” OR “children” OR “pediatric” OR “healthy children” OR “neurotypical”.

### 2.3 Eligibility criteria, study selection and data collection

Table 2 presents the eligibility criteria used to select articles in accordance with the Population, Exposure, Comparator, Outcome, and Study Design (PECOS) framework.

**Table 2:** Inclusion and exclusion criteria of study selection based on the PICOS framework.

PICOS framework	Inclusion	Exclusion
Population	Studies involving children aged 0–18 years	Studies involving adults with DS and ASD
Exposure	Studies involving children diagnosed with DS or ASD	Studies including children with co—occurring DS and ASD
Comparison	Studies reporting the outcomes of interest in DS vs. healthy controls and ASD vs. healthy controls	Studies on children with other neurodevelopmental disorders
Outcome	Studies reporting following outcome DFMT, dfmt, OHI-S, PI, GI	Studies reporting other outcomes on oral health
Study design	Case—control and cross—sectional with comparison group	RCT, review, case reports, congress abstracts, commentaries and editorials

The population criterion included studies involving children aged 0–18 years with DS and ASD**,** while studies focusing on adults with DS and ASD were excluded. Regarding exposure**,** only studies on children diagnosed with DS or ASD**,** were included. Studies involving children with co—occurring DS and ASD were excluded.

In terms of comparison, the review included studies that assessed outcomes in children with DS or ASD compared to healthy children. Studies that other neurodevelopmental disorders were excluded.

The outcome criteria focused on oral health indicators, including DFMT (dental caries experience in permanent teeth), dfmt (dental caries in primary teeth), OHI-S (Simplified Oral Hygiene Index), PI (Plaque Index), and GI (Gingival Index). Studies reporting other unrelated oral health outcomes were excluded.

Finally, eligible study designs included case–control and cross-sectional studies with a comparison group. Excluded study types were randomized controlled trials (RCT), reviews, case reports, conference abstracts, commentaries, and editorials.

The eligibility assessment and data collection were conducted in accordance with the PRISMA guidelines (31). Two independent reviewers (K.Zh. and N.B.) carried out a standardized literature search across all relevant databases. The search results were compiled in Mendeley, and duplicates were removed. Only unique records were then screened for relevance based on their titles and abstracts. In the next stage of eligibility assessment, full-text articles were reviewed against the inclusion and exclusion criteria outlined in Table 2, based on the PECOS framework. This review was carried out independently by both reviewers. Standardized data extraction forms were developed in advance, informed by preliminary search findings.

Data collection form included the following information: first author's last name, year of publication, country, study design, mean age of participants, neurodevelopmental condition (DS or ASD), comparator group (neurotypical children), and the reported mean values and standard deviations for oral health outcomes, including DFMT, dfmt, OHI-S, PI, and GI. Two independent data extraction sheets were compared and merged in the final stage. Any discrepancies in study selection or data interpretation were resolved through discussion with a third reviewer (N.J.), and full consensus was achieved for all included studies.

### 2.4 Meta-analysis

The meta-analysis of pooled mean differences was conducted using RStudio (version 2024.12.1.563) with R (version 4.3.2, released 2023-10-31) (32). We calculated pooled mean differences for five clinical indices (DMFT, dmft, OHI-S, PI, and GI) to compare outcomes between (1) children with DS and healthy controls, and (2) children with ASD and healthy controls. Analyses were performed using the *meta* and *metafor* packages. A random-effects model, based on the DerSimonian–Laird estimator, was employed to derive pooled mean differences with 95% confidence intervals. Forest plots were generated to visually represent the pooled mean differences. Heterogeneity was assessed using the *I*^2^ statistic, and potential moderating factors were investigated via meta-regression based on the year of publication. Sensitivity analyses were conducted using leave-one-out resampling to evaluate the robustness of the findings. Publication bias was assessed through funnel plot asymmetry and Egger's regression test for outcomes with at least ten studies. Finally, subgroup analyses were performed separately for DS vs. control and ASD vs. control comparisons to identify group-specific effects.

### 2.5 Risk of bias

The risk of bias (quality) assessment was conducted using the Mixed Methods Appraisal Tool (MMAT), which is specifically designed to appraise the methodological quality of qualitative, quantitative, and mixed-methods studies. For this review, we applied the quantitative descriptive criteria from the MMAT (version 2018) (33), appropriate for evaluating cross-sectional studies and case-control studies.

Each included study was assessed according to the following seven MMAT criteria**:**

1. Are the research questions well-defined?
2. Can the research questions be addressed using the data that has been gathered?
3. Does the sample plan adequately answer the research question?
4. Does the sample accurately reflect the intended audience?
5. Are the measurements accurate, valid, and trustworthy?
6. Is there little chance of nonresponse bias?
7. Can the research question be addressed by the statistical analysis?

Each criterion was rated as “Yes”, “No”, or “Can't tell” based on the reported methods and results in each article. Two reviewers independently performed the assessments after reaching consensus on the evaluation procedures. Any disagreements were resolved through discussion with a third reviewer.

Although the MMAT does not recommend computing an overall score, we report the total number of “Yes” ratings for transparency. Studies that met at least 5 out of 7 criteria with a “Yes” rating were considered to be of satisfactory quality and were included in the final synthesis. The detailed results of the quality assessment for all included studies are presented in Table 3.

**Table 3:** MMAT risk of bias assessment.

Author, year	Question 1	Question 2	Question 3	Question 4	Question 5	Question 6	Question 7	Total “Yes” score
Cross—sectional								
Habashneh, et al. (34)	Yes	Yes	Yes	Yes	Yes	Yes	Can’t tell	6
Carrada, et al. (35)	Yes	Yes	Yes	Yes	Yes	Yes	Can’t tell	6
Leiva-García, et al. (36)	Yes	Yes	Yes	Yes	Can't tell	Yes	Yes	6
Kuter, et al. (37)	Yes	Yes	Yes	Yes	Yes	Yes	Yes	7
Dharmadhikari, et al. (38)	Yes	Yes	Yes	Yes	Yes	Yes	Can't tell	6
Hamonari, et al. (39)	Yes	Yes	Yes	Yes	Yes	Yes	Yes	7
Case—control								
Lee, et al. (40)	Yes	yes	Yes	Yes	yes	yes	Can’t tell	6
Jaber, et al. (41)	Yes	Yes	Yes	Yes	Yes	Yes	Can’t tell	6
Areias, et al. (42)	Yes	Yes	Yes	Yes	Yes	Yes	Yes	7
Subramaniam, et al. (43)	Yes	Yes	Yes	Yes	Yes	Yes	Yes	7
Al—Maweri, et al. (44)	Yes	Yes	Yes	Yes	Yes	Yes	Yes	7
Khatib, et al. (45)	Yes	Yes	Yes	Yes	Yes	Yes	Yes	7
AlSarheed, et al. (46)	Yes	Yes	Yes	Yes	Yes	Yes	Yes	7
Fakroon, et al. (47)	Yes	Yes	Yes	Yes	Yes	Yes	Yes	7
Diab, et al. (48)	Yes	Yes	Yes	Yes	Yes	Yes	Yes	7
Ghaith, et al. (49)	Yes	Yes	Yes	Yes	Yes	Yes	Yes	7
Daneshvar, et al. (50)	Yes	Yes	Yes	Yes	Yes	Yes	Yes	7
Andreeva, et al. (51)	Yes	Yes	Yes	Yes	Yes	Yes	Yes	7
Bagattoni, et al. (52)	Yes	Yes	Yes	Yes	Yes	Yes	Yes	7
Moorthy, et al. (53)	Yes	Yes	Yes	Yes	Yes	Yes	Yes	7
Radzuan, et al. (54)	Yes	Yes	Yes	Yes	Yes	Yes	Yes	7
Narula, et al. (55)	Yes	Yes	Yes	Yes	Yes	Yes	Yes	7
Mahmood, et al. (56)	Yes	Yes	Yes	Yes	Yes	Yes	Can’t tell	6
Prynda, et al. (57)	Yes	Yes	Yes	Yes	Yes	Yes	Yes	7

### 2.6 Certainty of evidence evaluation

Adhering to the guidelines from the Cochrane Handbook for Systematic Reviews of Interventions, we assessed the certainty of evidence using the Grading of Recommendations Assessment, Development, and Evaluation (GRADE) framework (58). Furthermore, this assessment followed the procedures outlined in research notes on the evaluation of GRADE in systematic reviews (59). Certainty of evidence was calculated in RStudio, using the “GRADE” package. This framework comprises five domains: Risk of Bias, assessed using the MMAT scores mentioned above; Inconsistency, assessed via the *I*^2^ statistic; Indirectness, assessed via PECOS criteria; Imprecision, assessed by determining if the 95% CI of the pooled estimate crosses the threshold of interest; and Publication Bias, assessed using Egger's test results.

## 3 Results

### 3.1 Study selection and characteristics of the included studies

**Figure 1:**
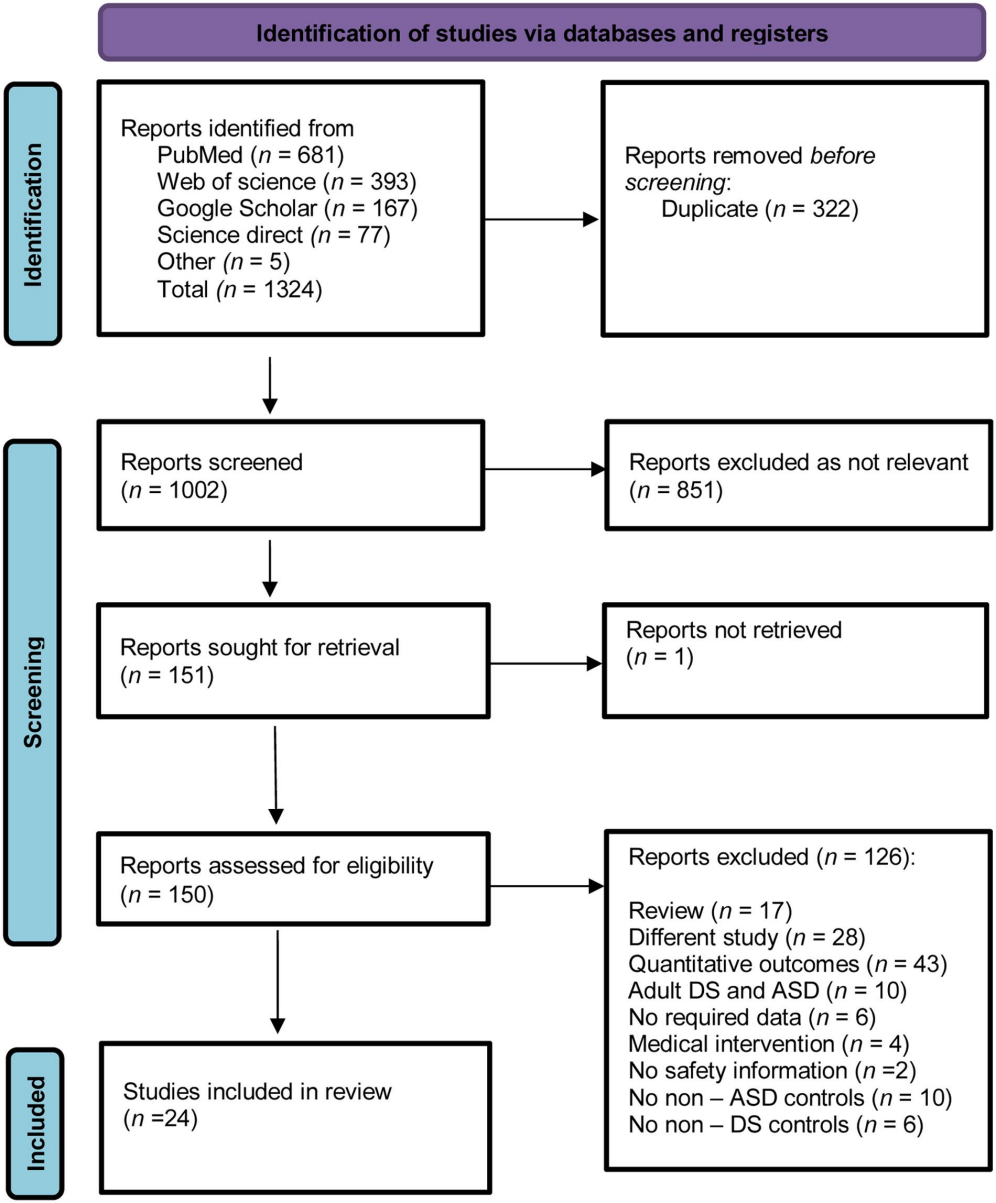
PRISMA flowchart of study inclusion.

A comprehensive search across PubMed, Web of Science, Google Scholar and ScienceDirect databases identified 1,324 records**.** After removing duplicates, 1,002 titles and abstracts were screened, of which 151 articles were selected for full-text evaluation. Following a full-text assessment, 24 studies met the PICOS eligibility criteria and were included in the meta-analysis. A total of 126 studies were excluded, including six that did not include healthy control groups for comparison with patients with DS. References for these six excluded studies are provided (60–63). The PRISMA flowchart illustrating the study selection and inclusion process is presented in Figure 1 (31).

The 24 included studies originated from a diverse range of countries. Ten studies focused on children with DS, while 14 investigated children with ASD, each compared to age-matched healthy control group. The majority of studies were conducted in Asia. In total, 527 children with DS, 1,221 children with ASD, and 1,875 controls were assessed. Participant ages ranged from 3 to 18 years**.** Various oral health indicators were collected from each study. Detailed study characteristics are presented in Table 4.

**Table 4:** Summary of included studies on oral health in children with DS and ASD compared with controls.

Last name, year	Design	Country	Age	Cases	Controls	Oral health data collected
Studies with DS patients						
Lee, et al. (40)	Case—control	Korea	8–17	19	41	OHI –S
Areias, et al. (42)	Case—control	Portugal	6–18	45	45	DFMT, dfmt
Habashneh, et al. (34)	Cross—sectional	Jordan	12–16	103	103	GI, DFMT
Subramaniam, et al. (43)	Case—control	India	7–12	34	34	DFMT, dfmt
AlSarheed et al. (46)	Case—control	Saudi Arabia	7–15	93	99	DFMT
Carrada, et al. (35)	Cross—sectional	Brazil	3–12	30	30	PI
Ghaith, et al. (49)	Case—control	United Arab Emirates	4–18	84	112	DFMT, dfmt, OHI
Andreeva, et al. (51)	Case—control	Bulgaria	3–15	60	60	PI
Dharmadhikari, et al. (38)	Cross—sectional	India	4–14	25	25	OHI –S
Hamonari, et al. (39)	Cross—sectional	Iraq	7–12	40	42	DFMT, dfmt, PI,GI
Studies with ASD patients						
Jaber, et al. (41)	Case—control	United Arab Emirates	6–18	61	61	DFMT,dfmt
Al—Maweri, et al. (44)	Case—control	Yemen	5–18	42	84	DFMT, PI,GI
Khatib, et al. (45)	Case—control	Egypt	3–13	100	100	DFMT, PI, GI
Fakroon, et al. (47)	Case—control	Libya	3–14	50	50	DFMT, dfmt
Diab, et al. (48)	Case—control	Saudi Arabia	4–15	50	50	PI, GI
Daneshvar, et al. (50)	Case—control	Iran	6–12	55	165	DFMT, dfmt
Leiva-García, et al. (36)	Cross—sectional	Spain	3–15	51	93	DFMT, dfmt
Kuter, et al. (37)	Cross—sectional	Turkey	5–16	285	122	DFMT, dfmt, PI
Bagattoni, et al. (52)	Case—control	Italy	M ± SD = 9.0 ± 2.9	64	64	DFMT, dfmt, PI
Moorthy, et al. (53)	Case—control	India	5–12	136	136	DFMT, dfmt, PI
Radzuan, et al. (54)	Case—control	Malaysia	3–16	2.8	30	DFMT, dfmt
Narula, et al. (55)	Case—control	India	5–14	80	80	DFMT, dfmt
Mahmood, et al. (56)	Case—control	Iraq	5–12	45	45	PI, GI
Prynda, et al. (57)	Case—control	Poland	3–12	74	74	OHI -S

ASD, autism spectrum disorder; DFMT, dental caries experience for permanent teeth; dfmt, dental caries experience for primary teeth; DS, down syndrome; GI, gingival index; OHI-S, simplified oral hygiene index; PI, plaque index.

## 4 Meta-analysis results

**Figure 2:**
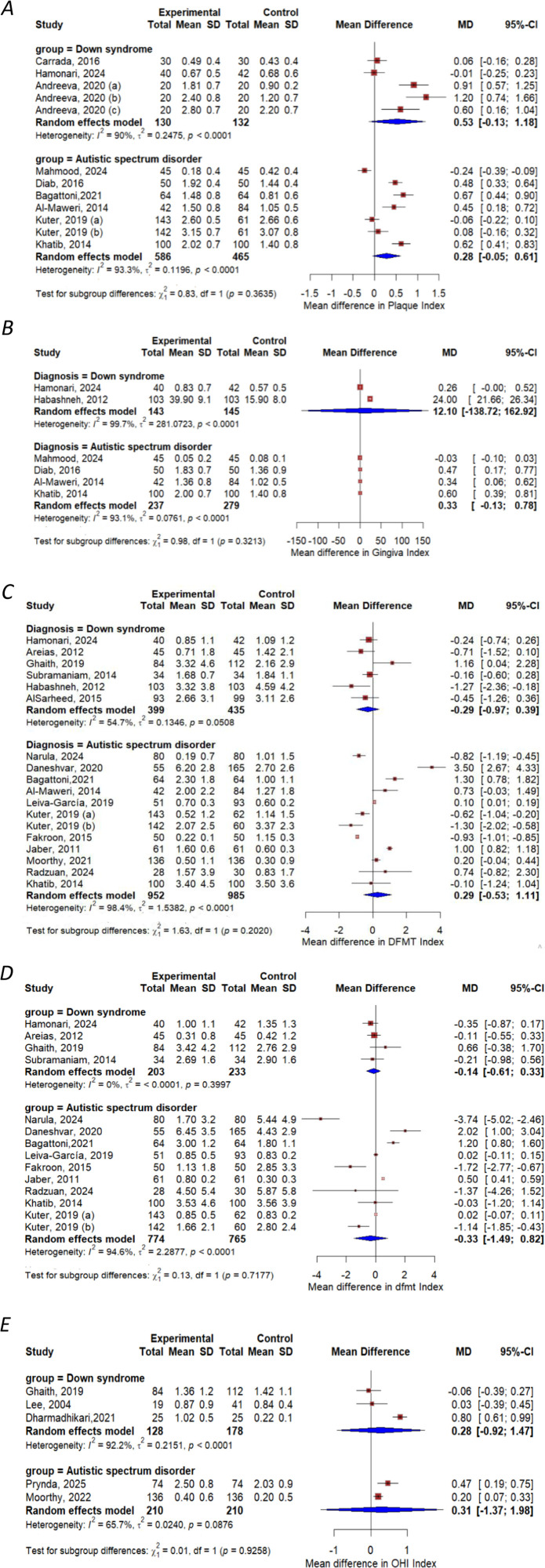
Meta-analysis of oral health Status among children with DS, ASD, and their healthy controls: **(A)** PI; **(B)** GI; **(C)** DFMT; **(D)** dfmt; **(E)** OHI – S. DFMT, dental caries experience for permanent teeth; dfmt, dental caries experience for primary teeth; OHI-S, simplified oral hygiene index; PI, plaque index; GI, gingiva index; DS, down syndrome; ASD, autism spectrum disorder*.* Group definitions: Kuter, 2019 **(A)**: 5–11 years (37)*;* Kuter, 2019 **(B)**: 12–16 years (37)*.* Andreeva, 2020 **(A)**: 3–6 years (51), Andreeva, 2020 **(B)**: 6–12 years (51), Andreeva, 2020 **(C)**: 12–14 years (51).

Figure 2 presents the pooled mean difference analysis of oral health indices among children with DS and ASD compared to healthy controls. Panel A illustrates the mean difference in PI scores between children with DS (five studies) and those with ASD (seven studies) relative to healthy peers. In the DS subgroup, the pooled mean difference was 0.53 (95% CI: −0.13–1.18), maintaining high heterogeneity (*I*^2^ = 90%, *p* < 0.0001), while in the ASD subgroup, the pooled mean difference in PI scores was 0.28 (95% CI: −0.05–0.61), with high heterogeneity (*I*^2^ = 93.3%, *p* < 0.0001).

Panel B shows the mean difference in GI between children with DS (two studies) and those with ASD (four studies) relative to healthy peers. In the DS subgroup, the pooled mean difference was 12.10 (95% CI: −0.138.72–162.92), maintaining heterogeneity (*I*^2^ = 99.7%, *p* < 0.0001), while in the ASD subgroup, the pooled mean difference in GI scores was 0.33 (95% CI: −0.13–0.78), with high heterogeneity (*I*^2^ = 93.1%, *p* < 0.0001).

Panel C shows the mean difference in DFMT index between children with DS (six studies) and those with ASD (twelve studies) relative to healthy peers. In the DS subgroup, the pooled mean difference was −0.29 (95% CI: −0.97–0.39), maintaining high heterogeneity (*I*^2^ = 54.7%, *p* < 0.05), while in the ASD subgroup, it was 0.29 (95% CI: −0.53–1.11), with high heterogeneity (*I*^2^ = 97.6%, *p* < 0.0001).

Panel D shows the mean difference in dfmt index between children with DS (four studies) and those with ASD (ten studies) relative to healthy peers. In the DS subgroup, the pooled mean difference was −0.14 (95% CI: −0.61–0.33), maintaining homogeneity (*I*^2^ = 0%, *p* < 0.39), while in the ASD subgroup, it was −0.33 (95% CI: −1.49–0.82), with high heterogeneity (*I*^2^ = 94.6%, *p* < 0.0001).

Panel E shows the mean difference in OHI-S index between children with DS (three studies) and those with ASD (two studies) relative to healthy peers. In the DS subgroup, the pooled mean difference was 0.28 (95% CI: −0.92–1.47), (*I*^2^ = 92.2%, *p* < 0.0001), while in the ASD subgroup, it was 0.31 (95% CI: −1.37–1.98), with heterogeneity (*I*^2^ = 65.7%, *p* < 0.08).

**Figure 3:**
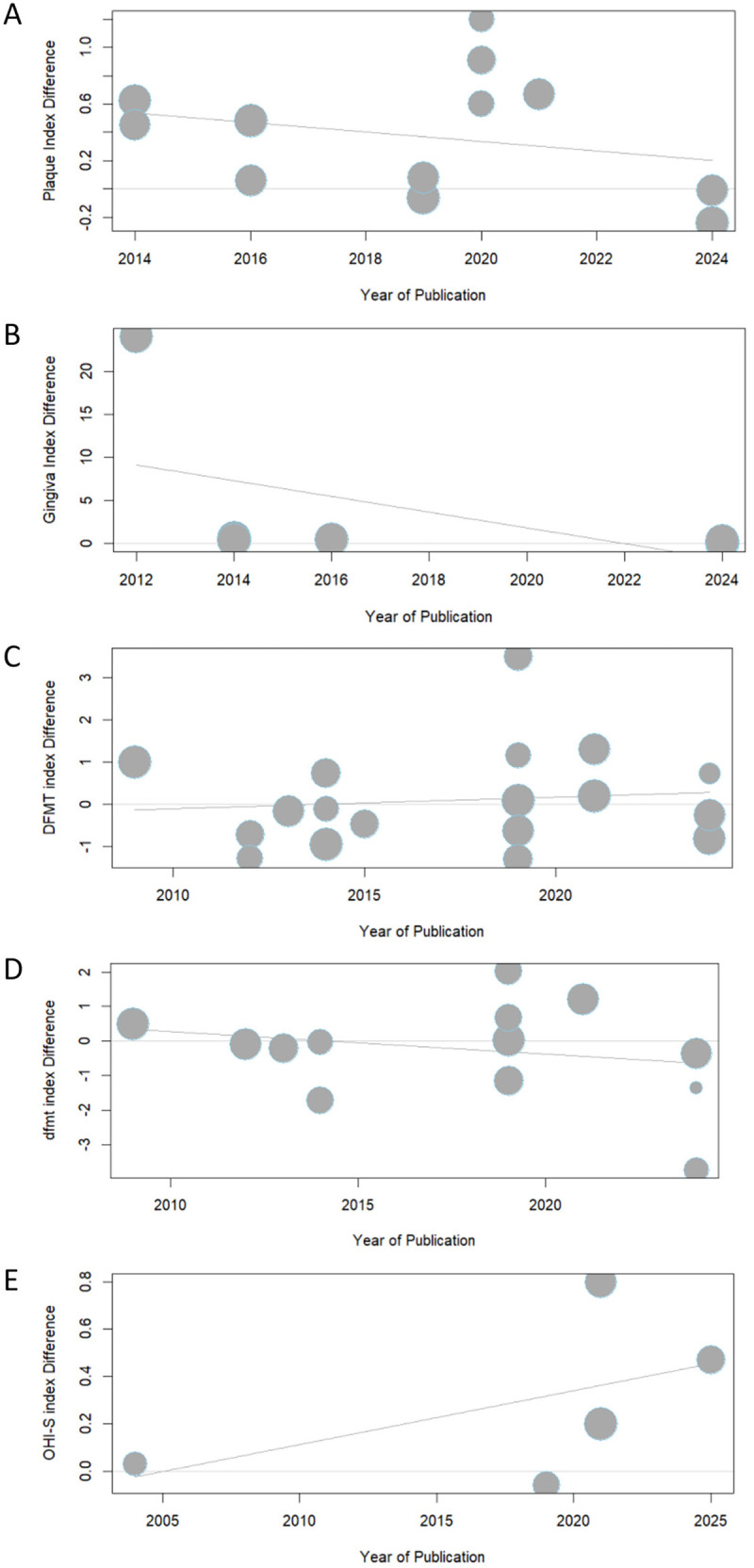
Meta-regression of the the oral health Status by the year of pubication among children with DS, ASD and healthy children **(A)** PI; **(B)** GI; **(C)** DFMT; **(D)** dfmt; **(E)** OHI – S. DFMT, dental caries experience for permanent teeth; dfmt, dental caries experience for primary teeth; OHI-S, simplified oral hygiene index; PI, plaque index; GI, gingiva index; DS, down syndrome; ASD, autism spectrum disorder.

The results of the meta-regression of oral health status among children with DS and ASD based on the year of publication are presented in Figure 3. In the meta-regression analysis, no significant associations were found between the year of publication and any of the oral health indices analyzed: (A) PI (*p* = 0.3991), (B) GI (*p* = 0.3075), (C) DMFT (*p* = 0.6679), (D) dmft (*p* = 0.4322), (E) OHI–S (*p* = 0.4005).

**Figure 4:**
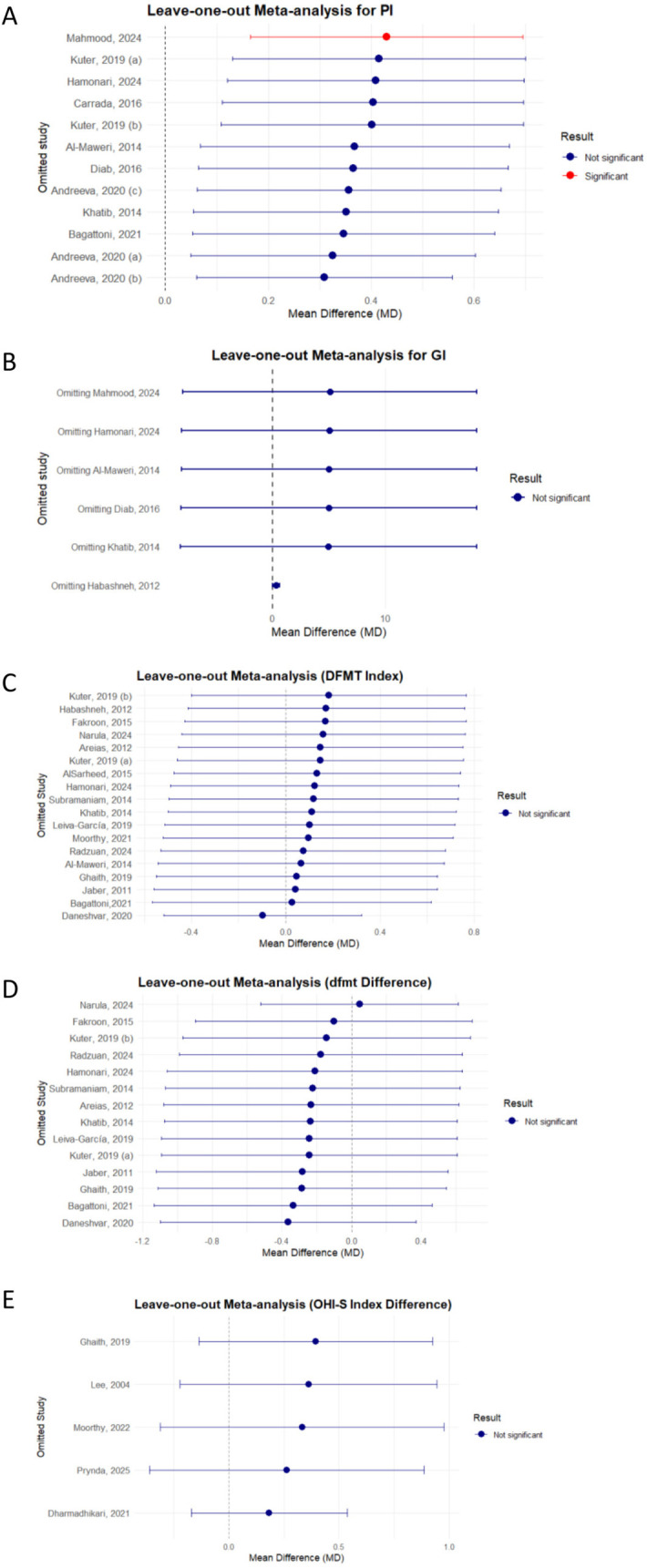
Sensitivity analysis of the oral health Status among children with DS, ASD and healthy children: **(A)** PI; **(B)** GI; **(C)** DFMT; **(D)** dfmt; **(E)** OHI – S. DFMT, dental caries experience for permanent teeth; dfmt, dental caries experience for primary teeth; OHI-S, simplified oral hygiene index; PI, plaque index; GI, gingiva index; DS, down syndrome; ASD, autism spectrum disorder. Group definitions: Kuter, 2019 **(A)**: 5–11 years (37); Kuter, 2019 **(B)**: 12–16 years (37). Andreeva, 2020 **(A)**: 3–6 years (51), Andreeva, 2020 **(B)**: 6–12 years (51), Andreeva, 2020 **(C)**: 12–14 years (51).

Sensitivity analyses are summarized in Figure 4. In Plot A (Plaque Index), one study, *Mahmood, 2024* (56)*,* showed a significant influence on the overall effect size. Its exclusion altered the effect to 0.43 (0.17–0.70), *p* = 0.0047, with heterogeneity reduced to 88.5%. In Plots B–E (Gingival Index, DMFT, dmft, OHI–S), no single study significantly impacted the overall results.

**Figure 5:**
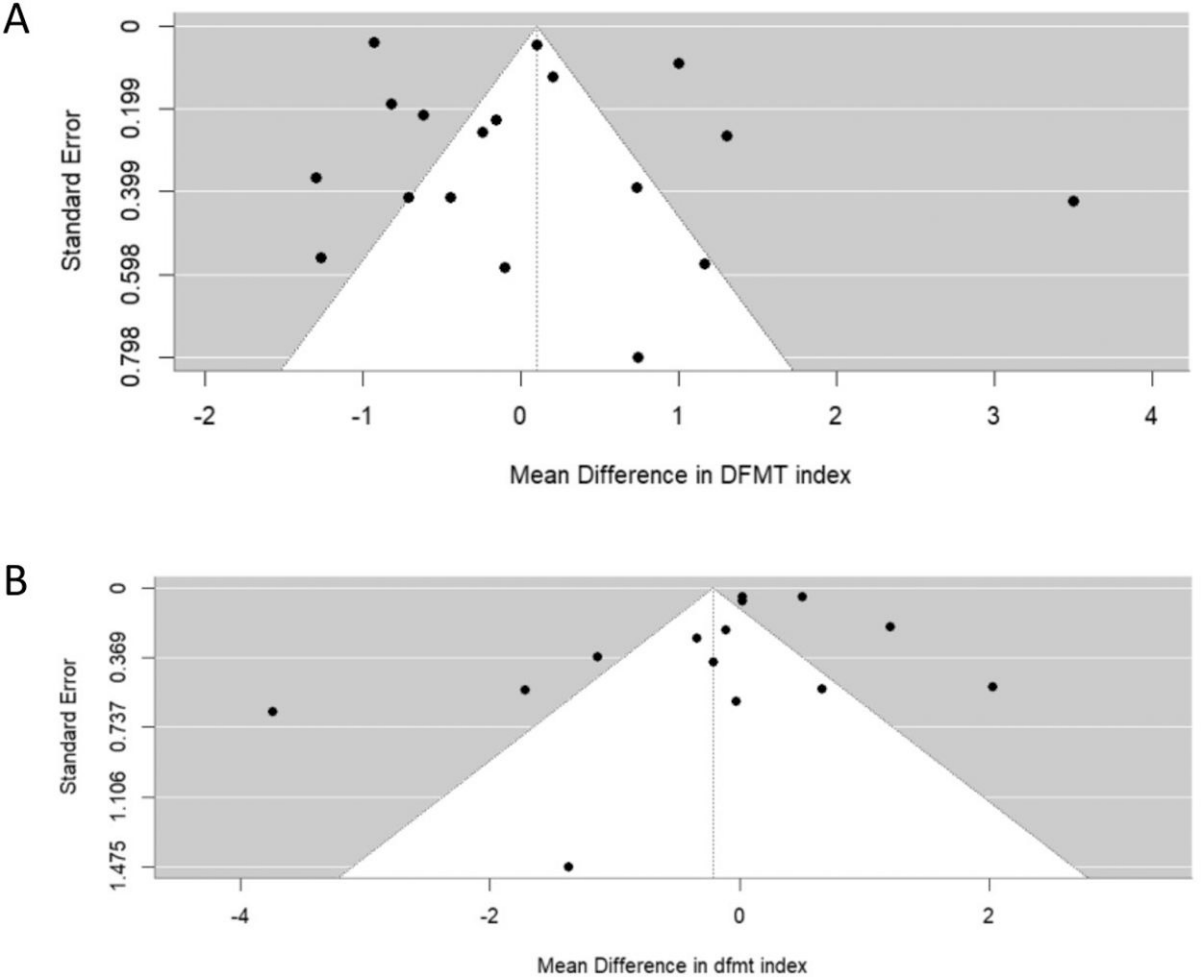
Publication bias assessment.

Publication bias assessment results are presented in Figure 5 (Panels A,B). For the DMFT and dmft indexes, the funnel plots appeared symmetrical, and Egger's test results were non-significant (*p* = 0.5466 and *p* = 0.1597, respectively), indicating no evidence of publication bias. However, for the PI, GI, and OHI-S indexes, fewer than 9 articles were included, and therefore, publication bias assessment was not performed.

The certainty of evidence assessment results for all pooled estimates are detailed in Table 5, based on the GRADE framework. The five meta-analytic outcomes incorporated data from case—control and cross—sectional analysis. Risk of bias was low across all outcomes. Inconsistency was judged “serious” for every outcome, whereas indirectness was considered “not serious.” Imprecision was serious for GI, DMFT, dmft, and OHI-S, but not for PI. Publication bias was not present for DMFT and dmft, while it was not assessed for PI, GI, and OHI-S because fewer than nine studies were available. Overall, the certainty of evidence was rated as low for all five pooled outcomes.

**Table 5:** Evaluation of the certainty of evidence using GRADE framework.

Outcome	Risk of Bias	Inconsistency	Indirectness	Imprecision	Publication Bias	Certainty of Evidence
MD in PI score	Low	Serious	Not serious	Not serious	Not assessed	Low
MD in GI score	Low	Serious	Not serious	Serious	Not assessed	Low
MD in DFMT score	Low	Serious	Not serious	Serious	No	Low
MD in dfmt score	Low	Serious	Not serious	Serious	No	Low
MD in OHI – S score	Low	Serious	Not serious	Serious	Not assessed	Low

ASD, autism spectrum disorder; DFMT, dental caries experience for permanent teeth; dfmt, dental caries experience for primary teeth; DS, down syndrome; GI, gingival index; MD, mean difference; OHI-S, simplified oral hygiene index; PI, plaque index.

## 5 Discussion

Our systematic review and meta-analysis yielded several key findings regarding oral health indices in children. First, contrary to our initial hypothesis that neurodevelopmental disorders would be associated with poorer oral health outcomes in children with DS and ASD, the results indicated no statistically significant differences compared with healthy peers for the following indices: GI, DMFT, dmft, and OHI-S. Second, sensitivity analyses largely confirmed the robustness of these findings, with the exception of one influential study affecting the PI estimates. Furthermore, no evidence of substantial publication bias was identified in analyses where formal assessment was feasible.

Our findings contribute to the broader literature on oral health disparities among children with neurodevelopmental conditions. Firouzeh N. et al. (64) previously reported significantly higher PI and GI scores in individuals with DS compared to healthy controls. In contrast, our subgroup analysis of children with DS did not reveal statistically significant differences in these indices, although the pooled estimate for the combined DS and ASD group did suggest elevated PI. One plausible explanation for this discrepancy may be the influence of age-related changes in oral hygiene behaviors and dental health outcomes, which tend to converge across populations as children mature. This interpretation is supported by prior studies indicating that DS does not significantly affect DMFT scores (8, 29). Similarly, Flávia et al. (11) found no significant difference in OHI between individuals with DS and healthy controls.

Regarding children with ASD, two meta-analyses have reported that, compared with their neurotypical peers, children with ASD tend to have poorer oral hygiene and greater susceptibility to dental caries (65, 66). Da Silva et al. (9) and Magdalena et al. (57) found that the prevalence of dental caries and periodontal disease in children and young adults with ASD can be considered high compared with healthy children. Similarly, Xiaoqin et al. (67) reported a higher mean DMFT, as well as higher PI and GI, in children with ASD compared to healthy controls suggesting that children with ASD have poorer oral hygiene, and higher risk of caries. Our findings showing no significant difference in GI, DMFT, dmft, or OHI-S scores contrast with some prior literature due to factors like the heterogeneity of included studies.

However, some studies have produced controversial results. For example, a few investigations reported lower caries prevalence in children with ASD compared to healthy controls (68, 69). Robertson et al. (70) reported no significant difference in dental caries levels between children with learning disabilities and their neurotypical peers, but subgroup analysis revealed lower caries levels in permanent teeth among children with DS and ASD. Such discrepancies may reflect differences in study populations, diagnostic criteria, dietary patterns, or preventive dental care.

Socioeconomic status also plays a critical role in shaping oral health outcomes. According to Costacurta et al. (71), healthy children under 12 years of age from families with very low income (<€6,000) exhibited significantly higher DMFT scores compared with those from higher-income households (>€20,000), underscoring the strong link between economic disadvantage and oral health. Cultural differences may also influence oral health outcomes. For instance, Zhang et al. (17) found that Asian children with ASD had significantly higher dmft scores compared with their non-Asian counterparts. This disparity may reflect the influence of cultural and socioeconomic determinants such as parental attitudes toward oral health, and societal stigma related to disability which collectively contribute to poorer oral health outcomes among Asian children with ASD (72).

In our study, the absence of significant differences across most oral health indicators for children with DS and ASD may reflect the complex interplay of multiple factors influencing oral health in these populations. These include caregiver involvement, socioeconomic conditions, access to dental services, and behavioral challenges. Furthermore, barriers to care—such as practitioners' limited confidence and specialized training, restricted access to specialist services, and inadequate behavioral or communication support during treatment may contribute to unmet oral health needs (73, 74).

This review has several limitations, including substantial heterogeneity, the overall low certainty of evidence, and reliance on case–control and cross-sectional study designs. Therefore the findings of our study should be interpreted with caution. The lack of statistically significant differences in some oral health indices between children with learning disabilities and neurotypical children does not eliminate the need for targeted interventions. Instead, it highlights persistent barriers to care. From a practical perspective, the observed disparities in PI indicators underscore the need to strengthen care delivery and address systemic barriers. Public health initiatives should focus on inclusive, community-based programs that integrate oral health education into family support systems, expand provider training in special care dentistry, and ensure equitable access to preventive and therapeutic services. These efforts are crucial to reducing oral health disparities and promoting long-term well-being among children with neurodevelopmental disorders.

Future research should focus on improving study design and evidence quality, particularly through longitudinal studies, and should examine how socioeconomic status, cultural background, and dentists' experience influence the evaluation of oral health indices.

This review found that children with DS and ASD generally tend to have no consistent differences in oral health indicators such as PI, GI, DMFT, dmft, or OHI-S scores. The absence of consistent differences in certain indices does not reduce the importance of addressing oral health needs in these populations. Public health strategies should prioritize inclusive oral health education, improved provider training, and equitable access to dental services to achieve better outcomes for children with neurodevelopmental disorders.

## Data Availability

The datasets presented in this study can be found in online repositories. The names of the repository/repositories and accession number(s) can be found in the article/Supplementary Material.

## References

[1] Chen L Wang L Wang Y Hu H Zhan Y Zeng Z Global, regional, and national burden and trends of down syndrome from 1990 to 2019. Front Genet. (2022) 13:908482. 10.3389/FGENE.2022.908482 35910218 PMC9337874

[2] Zeidan J Fombonne E Scorah J Ibrahim A Durkin MS Saxena S Global prevalence of autism: a systematic review update. Autism Res. (2022) 15:778–90. 10.1002/AUR.2696 35238171 PMC9310578

[3] Antonarakis SE Skotko BG Rafii MS Strydom A Pape SE Bianchi DW Down syndrome. Nat Rev Dis Prim. (2020) 6:9. 10.1038/S41572-019-0143-7 32029743 PMC8428796

[4] Lord C Elsabbagh M Baird G Veenstra-Vanderweele J. Autism spectrum disorder. Lancet (London, England). (2018) 392:508–20. 10.1016/S0140-6736(18)31129-2 30078460 PMC7398158

[5] Junnarkar VS Tong HJ Hanna KMB Aishworiya R Duggal M. Qualitative study on barriers and coping strategies for dental care in autistic children: parents’ perspective. Int J Paediatr Dent. (2023) 33:203–15. 10.1111/IPD.13035 36271894

[6] Allerton LA Welch V Emerson E. Health inequalities experienced by children and young people with intellectual disabilities: a review of literature from the United Kingdom. J Intellect Disabil. (2011) 15:269–78. 10.1177/1744629511430772 22129526

[7] Anders PL Davis EL. Oral health of patients with intellectual disabilities: a systematic review. Spec Care Dentist. (2010) 30:110–7. 10.1111/J.1754-4505.2010.00136.X 20500706

[8] da Silva MCPM Lyra MCA de Almeida HCR de Alencar Filho AV Heimer MV Rosenblatt A. Caries experience in children and adolescents with down syndrome: a systematic review and meta-analysis. Arch Oral Biol. (2020) 115:104715. 10.1016/j.archoralbio.2020.104715 32422361

[9] da Silva SN Gimenez T Souza RC Mello-Moura ACV Raggio DP Morimoto S Oral health status of children and young adults with autism spectrum disorders: systematic review and meta-analysis. Int J Paediatr Dent. (2017) 27:388–98. 10.1111/IPD.12274 27796062

[10] Silva DBE de Castro Corrêa C Weber SAT. Orofacial myofunctional and polysomnographic characteristics of children with down syndrome and obstructive sleep apnea: a pilot study. CoDAS. (2024) 36:e20230119. 10.1590/2317-1782/20242023119en 38808857 PMC11166037

[11] Scalioni FAR Carrada CF Tavares MC Abreu LG Ribeiro RA Paiva SM. Oral health characteristics in children and adolescents with down syndrome. Spec Care Dentist. (2024) 44:542–9. 10.1111/SCD.12883 37271587

[12] Sandeepa NC Al Hagbani S Alhammad F Al Shahrani A Al Asmari S. Oral health status of down’s syndrome patients in Aseer, Saudi Arabia. J Pharm Bioallied Sci. (2021) 13:S656–9. 10.4103/JPBS.JPBS_593_20 34447174 PMC8375836

[13] Shukla D Bablani D Chowdhry A Thapar R Gupta P Mishra S. Dentofacial and cranial changes in down syndrome. Osong Public Heal Res Perspect. (2014) 5:339–44. 10.1016/J.PHRP.2014.09.004 PMC4281609 25562042

[14] Boukhalfa Y Kraoua L Maazoul F Zaroui J M’rad R. Prevalence and characteristics of oral and dental anomalies in Tunisian individuals with down syndrome: a descriptive study. Egypt J Med Hum Genet. (2025) 26:1–8. 10.1186/s43042-025-00700-z

[15] Goud EVSS Gulati S Agrawal A Pani P Nishant K Pattnaik SJ Implications of down’s syndrome on oral health status in patients. J Fam Med Prim Care. (2021) 10:4247–52. 10.4103/JFMPC.JFMPC_885_21 PMC8797122 35136797

[16] Al-Sufyani GA Al-Maweri SA Al-Ghashm AA Al-Soneidar WA. Oral hygiene and gingival health status of children with down syndrome in Yemen: a cross-sectional study. J Int Soc Prev Community Dent. (2014) 4:82–6. 10.4103/2231-0762.139429 25254190 PMC4170549

[17] Zhang Y Lin L Liu J Shi L Lu J. Dental caries status in autistic children: a meta-analysis. J Autism Dev Disord. (2020) 50:1249–57. 10.1007/S10803-019-04256-X 32008179

[18] Nagda R Le T Lin B Tanbonliong T. Oral hygiene practice and home-care challenges in children with autism spectrum disorder in San Francisco: cross-sectional study. Spec Care Dent. (2024) 44:837–44. 10.1111/SCD.12922 PMC13193393 37700541

[19] Geelhoed EA Bebbington A Bower C Deshpande A Leonard H. Direct health care costs of children and adolescents with down syndrome. J Pediatr. (2011) 159:541–5. 10.1016/J.JPEDS.2011.06.007 21784457 PMC3858577

[20] Lai B Milano M Roberts MW Hooper SR. Unmet dental needs and barriers to dental care among children with autism spectrum disorders. J Autism Dev Disord. (2012) 42:1294–303. 10.1007/S10803-011-1362-2 21909827

[21] Lewis CW. Dental care and children with special health care needs: a population-based perspective. Acad Pediatr. (2009) 9:420–6. 10.1016/J.ACAP.2009.09.005 19945077 PMC2787477

[22] Uliana JC Del’ Agnese CC Antoniazzi RP Kantorski KZ. Autistic individuals have worse oral status than neurotypical controls: a systematic review and meta-analysis of observational studies. Clin Oral Investig. (2024) 28:137. 10.1007/S00784-024-05500-0 38321186

[23] Sami W Ahmad MS Shaik RA Miraj M Ahmad S Molla MH. Oral health statuses of children and young adults with autism spectrum disorder: an umbrella review. J Clin Med. (2023) 13:59. 10.3390/JCM13010059 38202066 PMC10780292

[24] Sarnat H Samuel E Ashkenazi-Alfasi N Peretz B. Oral health characteristics of preschool children with autistic syndrome disorder. J Clin Pediatr Dent. (2016) 40:21–5. 10.17796/1053-4628-40.1.21 26696102

[25] Martins M Mascarenhas P Evangelista JG Barahona I Tavares V. The incidence of dental caries in children with down syndrome: a systematic review and meta-analysis. Dent J. (2022) 10:205. 10.3390/DJ10110205 PMC9689859 36354650

[26] Mathias MF Simionato MR Guare RO. Some factors associated with dental caries in the primary dentition of children with down syndrome. Eur J Paediatr Dent. (2011) 12:37–42. 21434734

[27] Davidovich E Aframian DJ Shapira J Peretz B. A comparison of the sialochemistry, oral pH, and oral health status of down syndrome children to healthy children. Int J Paediatr Dent. (2010) 20:235–41. 10.1111/J.1365-263X.2010.01045.X 20536584

[28] Scalioni FAR Carrada CF Martins CC Ribeiro RA Paiva SM. Periodontal disease in patients with down syndrome: a systematic review. J Am Dent Assoc. (2018) 149:628–639.e11. 10.1016/J.ADAJ.2018.03.010 29779565

[29] Deps TD Angelo GL Martins CC Paiva SM Pordeus IA Borges-Oliveira AC. Association between dental caries and down syndrome: a systematic review and meta-analysis. PLoS One. (2015) 10:e0127484. 10.1371/JOURNAL.PONE.0127484 26086498 PMC4472226

[30] Lam PPY Du R Peng S McGrath CPJ Yiu CKY. Oral health status of children and adolescents with autism spectrum disorder: a systematic review of case-control studies and meta-analysis. Autism. (2020) 24:1047–66. 10.1177/1362361319877337 31931609

[31] Page MJ McKenzie JE Bossuyt PM Boutron I Hoffmann TC Mulrow CD The PRISMA 2020 statement: an updated guideline for reporting systematic reviews. Br Med J. (2021) 372:n71. 10.1136/BMJ.N71 33782057 PMC8005924

[32] Introducing RStudio Team—Posit. Available online at: https://posit.co/blog/introducing-rstudio-team/ (Accessed August 29, 2025)

[33] Hong QN Fàbregues S Bartlett G Boardman F Cargo M Dagenais P The mixed methods appraisal tool (MMAT) version 2018 for information professionals and researchers. Educ Inf. (2018) 34:285–91. 10.3233/EFI-180221

[34] Al Habashneh R Al-Jundi S Khader Y Nofel N. Oral health status and reasons for not attending dental care among 12- to 16-year-old children with down syndrome in special needs centres in Jordan. Int J Dent Hyg. (2012) 10:259–64. 10.1111/j.1601-5037.2012.00545.x 22335361

[35] Carrada CF Scalioni FAR Cesar DE Devito KL Ribeiro LC Ribeiro RA. Salivary periodontopathic bacteria in children and adolescents with down syndrome. PLoS One. (2016) 11:e0162988. 10.1371/JOURNAL.PONE.0162988 27727287 PMC5058504

[36] Leiva-García B Planells E Planells del Pozo P Molina-López J. Association between feeding problems and oral health status in children with autism spectrum disorder. J Autism Dev Disord. (2019) 49:4997–5008. 10.1007/S10803-019-04211-W 31489541

[37] Kuter B Guler N. Caries experience, oral disorders, oral hygiene practices and socio-demographic characteristics of autistic children. Eur J Paediatr Dent. (2019) 20:237–41. 10.23804/ejpd.2019.20.03.13 31489825

[38] Dharmadhikari P Thosar N Baliga S Rathi N. Comparative evaluation of salivary constituents and oral health status in children with down’s syndrome. Eur J Gen Dent. (2016) 5:90–4. 10.4103/2278-9626.179558

[39] Hamonari NH. Salivary markers of oxidative stress and their relation to periodontal and dental status among children with down syndrome. Cureus. (2024) 16:e73852. 10.7759/CUREUS.73852 39559432 PMC11573228

[40] Lee SR Kwon HK Song KB Choi YH. Dental caries and salivary immunoglobulin A in down syndrome children. J Paediatr Child Health. (2004) 40:530–3. 10.1111/J.1440-1754.2004.00457.X 15367146

[41] Jaber MA. Dental caries experience, oral health status and treatment needs of dental patients with autism. J Appl Oral Sci. (2011) 19:212–7. 10.1590/S1678-77572011000300006 21625735 PMC4234331

[42] Areias C Sampaio-Maia B de Lurdes Pereira M Azevedo Á Melo P Andrade C Reduced salivary flow and colonization by mutans streptococci in children with down syndrome. Clinics (Sao Paulo). (2012) 67:1007–11. 10.6061/CLINICS/2012(09)04 23018295 PMC3438238

[43] Subramaniam P Girish Babu K Mohan Das L. Assessment of salivary total antioxidant levels and oral health status in children with down syndrome. Spec Care Dentist. (2014) 34:193–200. 10.1111/SCD.12054 24188359

[44] Al-Maweri S Halboub E Al-Soneidar W Al-Sufyani G. Oral lesions and dental status of autistic children in Yemen: a case-control study. J Int Soc Prev Community Dent. (2014) 4:S199–203. 10.4103/2231-0762.149040 25625079 PMC4304059

[45] El Khatib AA El Tekeya MM El Tantawi MA Omar T. Oral health status and behaviours of children with autism spectrum disorder: a case-control study. Int J Paediatr Dent. (2014) 24:314–23. 10.1111/IPD.12067 24750459

[46] AlSarheed M. A comparative study of oral health amongst trisomy 21 children living in Riyadh, Saudi Arabia: Part 1 caries, malocclusion, trauma. Saudi Dent J. (2015) 27:220–3. 10.1016/J.SDENTJ.2015.03.003 26644758 PMC4642185

[47] Fakroon S Arheiam A Omar S. Dental caries experience and periodontal treatment needs of children with autistic spectrum disorder. Eur Arch Paediatr Dent. (2015) 16:205–9. 10.1007/S40368-014-0156-6 25385711

[48] Diab HM Motlaq SS Alsharare A Alshammery A Alshammery N Khawja SG Comparison of gingival health and salivary parameters among autistic and non-autistic school children in Riyadh. J Clin Diagn Res. (2016) 10:ZC110–3. 10.7860/JCDR/2016/23373.8692 27891471 PMC5121788

[49] Ghaith B Al Halabi M Khamis A Kowash M. Oral health status among children with down syndrome in Dubai, United Arab Emirates. J Int Soc Prev Community Dent. (2019) 9:232. 10.4103/JISPCD.JISPCD_396_18 31198694 PMC6559046

[50] Daneshvar SH Kavianfar A Masoomi SH Daneshvar MM. Comparison of oral health status and behaviors between children with autistic spectrum disorder and healthy children in Rasht City, Iran. Cumhur Dent J. (2020) 23:38–44. 10.7126/CUMUDJ.638071

[51] Andreeva R Atanasova S. Prevalence of periodontal diseases in children with down syndrome. J IMAB—Annu Proc. Sci Pap. (2020) 26:3383–6. 10.5272/JIMAB.2020264.3383

[52] Bagattoni S Lardani L D’Alessandro G Piana G. Oral health status of Italian children with autism spectrum disorder. Eur J Paediatr Dent. (2021) 22:243–7. 10.23804/ejpd.2021.22.03.12 34544255

[53] Moorthy L Dixit UB Kole RC Gajre MP. Dietary sugar exposure and oral health status in children with autism spectrum disorder: a case-control study. J Autism Dev Disord. (2022) 52:2523–34. 10.1007/S10803-021-05151-0 34218395

[54] Radzuan NFM Halim RA Noor E. Oral health disparities among children with autism spectrum disorder and typically developing peers: a case-control study at universiti teknologi mara. J Heal Transl Med. (2024) 27:97–102. 10.22452/JUMMEC.SP2024NO1.11

[55] Narula V Goswami M Juneja M Kumar G. Comparative evaluation of oral health Status and treatment needs of children with autism spectrum disorder: a cross-sectional study. Cureus. (2024) 16:e58663. 10.7759/CUREUS.58663 38774179 PMC11106220

[56] Mahmood SS Alzubaidee AF Hussein VM. Assessment of saliva oxidative stress biomarkers and gingival health status in a sample of high-functioning autistic children in erbil city. Cureus. (2024) 16:e73717. 10.7759/CUREUS.73717 39677204 PMC11646319

[57] Prynda M Pawlik AA Emich-Widera E Kazek B Mazur M Niemczyk W Oral hygiene status in children on the autism spectrum disorder. J Clin Med. (2025) 14:1868. 10.3390/jcm14061868 40142676 PMC11942761

[58] Schünemann HJ Oxman AD Higgins JP Vist GE Glasziou P Guyatt GH. Chapter 11: Presenting results and “summary of findings” tables. In: Higgins JPT Green S, editors. Cochrane Handbook for Systematic Reviews of Interventions Cochrane Book Series, the Cochrane Collaboration. Chichester: References—Scientific Research Publishing (2008). p. 335–69. Available online at: https://www.scirp.org/reference/referencespapers?referenceid=2330713 (Accessed July 25, 2025).

[59] Brennan SE Johnston RV. Research note: interpreting findings of a systematic review using GRADE methods. J Physiother. (2023) 69:198–202. 10.1016/J.JPHYS.2023.05.012 37277291

[60] Yerkibayeva Z Yermukhanova G Saduakassova K Menchisheva Y Abdukalikova D Abu Z. Oral health status in children with autism spectrum disorder in Almaty, Kazakhstan. Res Sq. (2025). 10.21203/RS.3.RS-6682941/V1

[61] Subramaniam P Gupta M. Oral health status of autistic children in India. J Clin Pediatr Dent. (2011) 36:43–8. 10.17796/JCPD.36.1.L6287842UJ536X13 22900443

[62] Piraneh H Gholami M Sargeran K Shamshiri AR. Oral health and dental caries experience among students aged 7–15 years old with autism spectrum disorders in Tehran, Iran. BMC Pediatr. (2022) 22:1–12. 10.1186/s12887-022-03178-5 35248005 PMC8897865

[63] Hegde SK Ranjit O Bhat SS Kalal BS. Analysis of dental caries experience and parents perception on the oral health status of children with autism spectrum disorders from south India. Med J Bakirkoy. (2024) 20:189–95. 10.4274/BMJ.GALENOS.2024.2022.3-7

[64] Nilchian F Mosayebi N Tarrahi MJ Pasyar H. Comparison of oral indices in patients with down syndrome and healthy individuals: a meta-analysis study. Dent Res J (Isfahan). (2023) 20:104. 10.4103/drj.drj_113_23 38020256 PMC10680071

[65] Asiri FY Tennant M Kruger E. Oral health status of children with autism spectrum disorder in KSA: a systematic review and meta-analysis. J Taibah Univ Med Sci. (2024) 19:938. 10.1016/J.JTUMED.2024.09.005 39397870 PMC11470289

[66] da Motta TP da Mota DG Bitencourt FV Jardim PF Abreu LG Zina LG Dental caries of individuals with autism spectrum disorder (ASD): a systematic review and meta-analysis. J Autism Dev Disord. (2025):938. 10.1007/S10803-025-06754-7 39976759

[67] Pi X Liu C Li Z Guo H Jiang H Du M. A meta-analysis of oral health status of children with autism. J Clin Pediatr Dent. (2020) 44:1–7. 10.17796/1053-4625-44.1.1 31995423

[68] Du RY Yiu CKY King NM Wong VCN McGrath CPJ. Oral health among preschool children with autism spectrum disorders: a case-control study. Autism. (2015) 19:746–51. 10.1177/1362361314553439 25432504

[69] Jin Q He Z Xu D Lin R Zhang T Lv B Autism spectrum disorders and childhood caries: a comprehensive Mendelian randomization study. BMC Pediatr. (2025) 25:484. 10.1186/S12887-025-05839-7 40597832 PMC12220140

[70] Robertson MD Schwendicke F De Araujo MP Radford JR Harris JC McGregor S Dental caries experience, care index and restorative index in children with learning disabilities and children without learning disabilities; a systematic review and meta-analysis. BMC Oral Health. (2019) 19:146. 10.1186/S12903-019-0795-4 31307444 PMC6632188

[71] Costacurta M Epis M Docimo R. Evaluation of DMFT in paediatric patients with social vulnerability conditions. Eur J Paediatr Dent. (2020) 21:70–3. 10.23804/EJPD.2020.21.01.14 32183533

[72] Li Q Wang Y Knight JC Yi Y Ozbek S Shariati M Dental health status, dentist visiting, and dental insurance of Asian immigrants in Canada. Int J Equity Health. (2023) 22:73. 10.1186/S12939-023-01863-0 37098603 PMC10131415

[73] Da Rosa SV Moysés SJ Theis LC Soares RC Moysés ST Werneck RI Barriers in access to dental services hindering the treatment of people with disabilities: a systematic review. Int J Dent. (2020) 2020:9074618. 10.1155/2020/9074618 32774378 PMC7396116

[74] Ma Y Lee LY Zhang X. Affiliate stigma and related factors among parents of autism spectrum condition: a pilot study from mainland China. Autism Dev Lang Impair. (2023) 8:23969415231168570. 10.1177/23969415231168567 PMC10090549 37064167

