# Supplementary material for: Oral hygiene and caries experience in children with down syndrome and autism spectrum disorder: a systematic review and meta-analysis
Source: Front Dent Med. 2026 Jan 23;6:1726952. doi: 10.3389/fdmed.2025.1726952 (PMC12876141; doi:10.3389/fdmed.2025.1726952)
Supplement: Supplementary file 1 [file Table1.docx]

**Search strategy of the Systematic Review**

| **Pubmed** | **Web of Science** | **Science Direct/or other** | Google Scholar |
| --- | --- | --- | --- |
| **Criteria: Search in All Fields.**  **Filter: Language: English, Russian , Human**  **Search date: May 2025**  **N - 681**  (("Oral Health"[Mesh] OR "Oral Hygiene"[Mesh] OR "Dental Caries" OR **"**Tooth Decay**"** [Mesh] OR "Oral Status" OR "Oral Hygiene Index" OR "Plaque Index" OR "Gingival Index" OR "Silness and Loe" OR "OHI-S" OR "DMFT" OR **"**DMFS**"**))  AND  (("Down Syndrome"[Mesh] OR "Trisomy 21" OR "Down syndrome children")  OR  ("Autism Spectrum Disorder"[Mesh] OR "Autism" OR "Autistic children")  ("Healthy children" OR "Neurotypical children"))  AND  ("Child"[Mesh] OR "Pediatric" OR "Children") | **Criteria: Search in All Fields.**  **Filter: Language: English, Russian**  **Search date: May 2025**  **N-393**  (("oral health" OR "oral hygiene" OR "dental health" OR "dental status" OR "tooth decay" OR "oral status" OR "Dental Caries") AND ("Plaque Index" OR "Gingival Index" OR "Oral Hygiene Index" OR "OHI" OR "OHI-S" OR "DMFT" OR "DMFS" OR "Dental Caries") AND ("Down Syndrome" OR "Trisomy 21" OR "Autism" OR "Autism Spectrum Disorder" OR "Autistic children" OR "Healthy children" OR "Typically developing children" OR "Neurotypical children") AND ("children" OR "pediatric")) | **Criteria: Search in All Fields.**  **Filter: Language: English Search date: May 2025**  **N-77**  ("oral health" OR "oral hygiene" OR "dental caries" OR "tooth decay" OR "DMFT" OR "DMFS")  AND  ("Down syndrome" OR "trisomy 21" ) – 38  ("oral health" OR "oral hygiene" OR "dental caries" OR "tooth decay" OR "DMFT" OR "DMFS")  AND  ("Autism" OR "Autism Spectrum Disorder" OR "Autistic children") – 40 | **Criteria: Search in Title only Fields.**  **Search date: May 2025**  **Filter: Language: English**  **N-167**  "oral health" OR "oral hygiene" OR "dental caries" OR "tooth decay" OR "plaque index" OR "gingival index" OR "OHI-S" OR "DMFT" OR "DMFS" "Down syndrome" OR "Trisomy 21" OR "Autism" OR "Autism spectrum disorder" OR "Healthy children" OR "Neurotypical children" Children OR Pediatri "oral health" OR "oral hygiene" OR "dental caries" OR "tooth decay" OR "plaque index" OR "gingival index" OR "OHI S" OR DMFT OR DMFS OR "Down syndrome" OR "Trisomy 21" OR Autism OR "Autism spectrum disorder" OR "Healthy children" OR "Neurotypical children" OR Children OR PediatriS" OR "DMFT" OR "DMFS")  AND  ("Down syndrome" OR "Trisomy 21" OR "Autism" OR "Autism spectrum disorder" OR "Healthy children" OR "Neurotypical children")  AND  (Children OR Pediatric) |
